# Relationship between obesity indicators and hypertension–diabetes comorbidity in an elderly population: a retrospective cohort study

**DOI:** 10.1186/s12877-023-04510-z

**Published:** 2023-11-30

**Authors:** Haojie Li, Zhan Shi, Xuejiao Chen, Junjie Wang, Jiacheng Ding, Shuoji Geng, Xinyuan Sheng, Songhe Shi

**Affiliations:** 1 College of Public Health, Zhengzhou University, Zhengzhou, Henan China; 2 Department of Pharmacy, Zhengzhou People’s Hospital, Zhengzhou, Henan China

**Keywords:** Hypertension-diabetes comorbidity, Body mass index, Waist circumference, Waist-to-height ratio, Cohort, Elderly

## Abstract

**Background:**

The prevalence of obesity, hypertension and diabetes is increasing. Hypertension and diabetes are common complications. Additionally, obesity and hypertension–diabetes comorbidity (HDC) are both closely related to insulin resistance. The aim of this study was to determine the association of obesity indicators with HDC in elderly individuals.

**Methods:**

This retrospective cohort study included 74,955 subjects aged ≥ 60 years living in Xinzheng, Henan Province, from January 2011 to December 2019. The data were collected from the annual health examination dataset. Cox proportional hazard regression models and competing-risk survival regression models were used to examine the relationships between the three indicators and HDC risk.

**Results:**

After 346,504 person-years of follow-up, HDC developed in 9,647 subjects. After further adjustments for confounders and death competing risks, compared with a body mass index (BMI) of 18.5–23.9 kg/m^2^, the fully adjusted hazard ratios (HRs) and 95% confidence intervals (CIs) of BMI < 18.5, 24–27.9 and ≥ 28 kg/m^2^ for HDC morbidity were 0.651(0.538,0.788),1.00,1.396(1.336,1.459) and 1.769(1.666,1.878), respectively. Moreover, participants with abdominal obesity measured via waist circumference (WC) or waist-to-height ratio (WtHR) had a higher risk of HDC (HR:1.513; 95% CI: 1.45,1.578 and HR:1.412;95% CI: 1.353,1.473), respectively, than participants with low WC or with low WtHR. In the joint analyses, the highest risk was observed in participants who were overweight and who had central obesity (HR: 1.721; 95% CI: 1.635, 1.811) compared with the nonoverweight and noncentral obesity groups.

**Conclusions:**

Increased BMI, WC and WtHR were associated with an increased risk of HDC. There was an additive interaction between general body adiposity (as measured via BMI) and central obesity (as measured via WC and WtHR) for HDC. Therefore, reasonable control of BMI, WC and WtHR may be an effective measure to prevent HDC among elderly individuals.

**Supplementary Information:**

The online version contains supplementary material available at 10.1186/s12877-023-04510-z.

## Introduction

Obesity has become a major challenge to the health of the population in China. China has the highest number of people who are overweight and who have obesity throughout the world, and the fast-rising prevalence of obesity has placed a considerable burden on the country’s health-care system [1–3]. Based on Chinese criteria [4], the most recent national data showed that 16.4% of Chinese adults had obesity and another 34.3% were overweight [3]. With rapid socioeconomic development and increasingly obesogenic environments, this increasing trend is unlikely to be reversed in the near future. Strong evidence from prospective cohort studies has demonstrated significant adverse associations between being overweight and obesity and major noncommunicable diseases, such as hypertension, diabetes mellitus, and certain cancers [5–8].

Diabetes mellitus and hypertension are among the most common diseases and cardiovascular risk factors, respectively [9]. Hypertension and diabetes are common complications of various disorders. Hypertension is reported in over two-thirds of patients with type 2 diabetes, and its development coincides with the development of hyperglycemia [10]. Additionally, greater than 50% of patients with diabetes mellitus, (either type 1 or 2), ultimately develop hypertension as a complication [11]. Hypertension–diabetes comorbidity (HDC) refers to the coexistence of the two diseases. Accumulating evidence suggests that the development of hypertension and diabetes mellitus corresponds to each other over time, and insulin resistance (IR) is a common feature of both prediabetes and prehypertension and an antecedent of progression to two respective disease states [12]. Therefore, HDC is closely related to IR [13].

General obesity and obesity in the upper body and abdomen are associated with an increased risk of IR, and obesity is the main cause of IR [14]. Therefore, obesity is likely correlated with HDC. Body mass index (BMI) and waist circumference (WC) are two common anthropometric measures of obesity in clinical and public health practice. Recently, the waist-to-height ratio (WtHR) has been used as a predictive factor for incident hypertension and diabetes in several studies [15, 16]. The maintenance of BMI, WC and WtHR to a normal range may be an important strategy to significantly reduce the occurrence of IR and subsequent metabolic diseases [14, 17].

To our knowledge, many studies have been restricted to either the assessment of general obesity or central obesity, as well as being confined to either hypertension or diabetes, and few researchers have examined the effects of general and central obesity on the risk of HDC. Moreover, the relationship between obesity and HDC was also reported to be different in different races and countries [13, 18]. In addition, the cutoffs of BMI and WC for evaluating systemic obesity and abdominal obesity are different in China and Western countries.

Therefore, the relationship between obesity indicators (BMI, WC and WtHR) and HDC should be further studied in the Chinese population. Thus, this study was conducted to assess the independent association of BMI, WC and WtHR with HDC, as well as their possible additive interactions on the risk of HDC for the elderly population by using a large and contemporary population in central China.

## Methods

### Study design and study population

The present study was a population-based, retrospective cohort study. The subjects of this study were older people in Xinzheng, Henan Province, Central China. Data originated from the annual residents’ health records. The electronic health records of residents mainly included three components: questionnaire surveys and anthropometric and laboratory measurements. From January 2011 to December 2019, the study cohort included 100,724 subjects aged over 60 years with at least two physical examination data points. From the 100,724 participants, we initially excluded 14,895 participants who had HDC at baseline. Subsequently, we excluded incomplete baseline data on smoking, drinking, physical activity, resting heart rate (RHR), BMI, WC and WtHR (*n* = 10,874). Finally, we had 74,955 participants who were used to assess the risk of HDC with BMI, WC and WtHR (Supplemental Fig. 1). Supplemental Table 1 descripted follow-up of the study population and the rate of complete cases and death every year. The onset time of HDC was defined as the time of physical examination when the HDC was observed. This study was approved by the Ethics Committee of Zhengzhou University (Reference Number: ZZUIRB2019-019), and written informed consent was obtained from all of the participants.

### Baseline examination and data collection

All of the participants completed a standardized questionnaire that included their sociodemographic characteristics (age, gender and marital status), medical history (hypertension, diabetes, coronary heart disease and stroke, among other disorders), smoking, drinking and physical activity. Based on marital status, smoking and drinking statuses, participants were classified as follows: living with a partner or without a partner; nonsmokers or previous/current smokers; and never, occasional, frequent, or daily drinkers. Physical activity was classified as occurring never, occasionally, more than once a week or daily. Physical examinations were conducted by uniformly trained investigators via a standard protocol. Participants were asked to maintain a standing position while wearing light clothes without shoes and were measured twice. Subsequently, the average value was recorded. Additionally, height and weight were measured to the nearest 0.1 cm and 0.1 kg, respectively, and waist circumference was measured midway between the lower edge of the costal arch and the upper edge of the iliac crest to the nearest 0.1 cm under standardized conditions following a standard protocol. BMI was calculated as weight(kg) divided by height squared (m), and WtHR was determined via WC (cm) divided by height (cm). Moreover, resting heart rate (RHR) and blood pressure were measured twice after subjects had rested for at least 5 min in a seated position by using an automatic sphygmomanometer (Omron HEM-7125, Kyoto, Japan). Blood samples were obtained after an overnight fast of at least 8 h and were measured by using an automatic biochemical analyzer (DIRUICS380, Changchun, China).

BMI was analyzed via the following 2 methods. 1) BMI was measured as the following four groups according to the BMI classification standard for Chinese individuals [3]: underweight (BMI < 18.5 kg/m^2^), normal (18.5–23.9 kg/m^2^), overweight (24–27.9 kg/m^2^) and obese (≥ 28 kg/m^2^), and 2) as a continuous variable. WC was measured at the midpoint of the distance between the lowest costal ridge and the upper border of the iliac crest and was analyzed via the following 2 methods: 1) as a binary variable [3, 19] (normal waist: < 85 cm in females and < 90 cm in males; central obesity: ≥ 85 cm in females and ≥ 90 in males), and 2) as a continuous variable. WtHR was analyzed via the following 2 methods: 1) as a binary variable [20] (normal WtHR: < 0.5; central obesity ≥ 0.5)) and 2) as a continuous variable. In joint effect analysis, both WC < 85 cm in females and WC < 90 cm in males and WtHR < 0.5 were defined as noncentral obesity; either WC ≥ 85 cm in females and WC ≥ 90 in males or WtHR ≥ 0.5 was defined as central obesity.

### Outcome definition

In this study, diabetes was defined as (1) self-reported doctor-diagnosed diabetes, (2) fasting plasma glucose (FPG) ≥ 7.0 mmol/L and glycosylated hemoglobin (HbA1c) ≥ 6.5% or (3) current treatment with antidiabetic medication. Hypertension was defined as (1) self-reported doctor diagnosed hypertension, (2) systolic blood pressure (SBP) ≥ 140 mmHg or diastolic blood pressure (DBP) ≥ 90 mmHg or (3) current treatment with antihypertensive drugs. The outcomes of interest in this study included HDC morbidity and all-cause death from January 2011 to December 2019. Information on deaths was collected from the Centers for Disease Control’s cause of death reporting system in Xinzheng.

### Statistical analysis

Continuous variables were described as the means and standard deviations (SDs). Categorical variables are presented as numbers and proportions. The chi-square test for categorical variables and the Kruskal–Wallis test for continuous variables were used to compare the mean levels of baseline variables between subjects with and without HDC. The associations of BMI, WC and WtHR with the cumulative incidence of HDC were analyzed via a Cox proportional hazard regression model and a competing-risk survival regression model, and HRs with 95% CIs of BMI, WC and WtHR were expressed in separate models. Model 1 adjusted for age and sex. Model 2 adjusted for variables in Model 1 in addition to marital status, smoking, drinking, physical activity and RHR. Model 3 was analyzed by competing-risks survival regression adjusted covariates that were the same as Model 2. We used restricted cubic splines with four knots in the Cox models to characterize the dose–response association and to test whether there was a nonlinear association of the three indicators with HDC risk. In restricted cubic spline analysis, we selected knots according to Akaike information criterion (AIC) and knots’ positions were the medians of the corresponding variables(Supplemental Table 2). In addition, a stratified analysis was performed by sex subgroup via a Cox regression model to test the consistency of these relationships. The proportional hazards assumption was verified with graphical methods and with models including time-by-covariate interactions. Finally, the combined effect of BMI (≥ 24 kg/m^2^ or < 24 kg/m^2^), WC (≥ 90 cm in males/ ≥ 85 cm in females or < 90 cm in males/ < 85 cm in females) and WtHR (≥ 0.5 or < 0.5) was explored. We evaluated the existence of additive interactions by calculating the relative excess risk due to interaction (RERI), attributable proportion due to interaction (AP) and synergy index (S). Three sensitivity analysis were conducted to assess the robustness of the results. Statistical analyses were performed by using R software (version 4.2.2). *P* < 0.05 with two-sided tests was considered to indicate statistical significance.

## Results

### Baseline characteristics of the study participants

The general characteristics of the participants at baseline are described in Table 1. Data were analyzed for 74,955 elderly Chinese people (mean age: 66.68 years; standard deviation [SD]: 7.39). After 346,504 person-years of follow-up, HDC developed in 9647 participants; additionally, the overall incidence of HDC was 27.84/1,000 person-years. Moreover, 8,859 (11.82%) all cause deaths occurred, 8,033(90.68%) of which occurred before the onset of HDC. There were statistically significant differences in sex, smoking, drinking, physicial activity, RHR, BMI, WC and WtHR among patients with HDC compared to those without HDC (all *P* < 0.001).

**Table 1:** Baseline characteristics of the study population with and without HDC

Characteristics	Total (*n* = 74,955)	Non-HDC (*n* = 65,308)	HDC (*n* = 9,647)	*P* value
Age (years)	66.68 (7.39)	66.75 (7.50)	66.20 (6.60)	< 0.001
Gender (%)				< 0.001
Men	35869 (47.85)	31677 (48.50)	4192 (43.45)	
Women	39086 (52.15)	33631 (51.50)	5455 (56.55)	
Marital status (%)				0.006
Living with partner	60363 (80.53)	52494 (80.38)	7869 (81.57)	
Living without partner	14592 (19.47)	12814 (19.62)	1778 (18.43)	
Smoking (%)				< 0.001
Never	63934 (85.30)	55584 (85.11)	8350 (86.56)	
Current or previous	11021 (14.70)	9724 (14.89)	1297 (13.44)	
Drinking (%)				< 0.001
Never	69561 (92.80)	60650 (92.87)	8911 (92.37)	
Occasionally	3113 (4.15)	2742 (4.20)	371 (3.85)	
Frequently	683 (0.91)	601 (0.92)	82 (0.85)	
Daily	1598 (2.13)	1315 (2.01)	283 (2.93)	
Physicial activity (%)				< 0.001
Never	56621 (75.54)	49511 (75.81)	7110 (73.70)	
Occasionally	3605 (4.81)	3083 (4.72)	522 (5.41)	
More than once a week	3050 (4.07)	2553 (3.91)	497 (5.15)	
Daily	11679 (15.58)	10161 (15.56)	1518 (15.74)	
BMI(kg/m^2^)				< 0.001
Normal	38544 (51.42)	34382 (52.65)	4162 (43.14)	
Underweight	1529 (2.04)	1421 (2.18)	108 (1.12)	
Overweight	26528 (35.39)	22644 (34.67)	3884 (40.26)	
Obesity	8354 (11.15)	6861 (10.51)	1493 (15.48)	
WC(cm)				< 0.001
Normal waist	51483 (68.69)	45501 (69.67)	5982 (62.01)	
Central obesity	23472 (31.31)	19807 (30.33)	3665 (37.99)	
WtHR				< 0.001
Normal	30896 (41.22)	27481 (42.08)	3415 (35.40)	
Central obesity	44059 (58.78)	37827 (57.92)	6232 (64.60)	
RHR(beats/min)	73.59 (8.14)	73.48 (8.18)	74.33 (7.86)	< 0.001

*Abbreviations*: *BMI* body mass index, *WC* waist circumference, *WtHR* waist-to-height ratio, *RHR* resting heart rate, *HDC* hypertension–diabetes comorbidity

### Risk of HDC by baseline BMI, WC and WtHR

Table 2 shows the multivariate association between three indicators (BMI, WC and WtHR) and the risk of HDC morbidity. After further adjustments for confounders and death competing-risks (Model 2 and Model 3), the associations remained significant. In competing-risks Model 3, compared with BMI of 18.5–23.9 kg/m^2^(reference group), the fully HRs and 95% CIs of BMI < 18.5, 24–27.9 and ≥ 28 kg/m^2^ for HDC morbidity were 0.651(0.538,0.788),1.00,1.396(1.336,1.459) and 1.769(1.666,1.878), respectively (*P* < 0.001).Moreover, participants with abdominal obesity (WC ≥ 85 cm [female]/90 cm [male]) or high WtHR (≥ 0.5)had a 51.3% (95% CI: 1.45,1.578) and a 41.2% (95% CI: 1.353,1.473) higher risk of HDC, respectively, compared to participants with low WC (< 85 cm [female]/90 cm [male]) or with low WtHR (< 0.5).

**Table 2:** Risk of HDC by baseline BMI, WC and WtHR

Variables	No. of cases	No. of person-years	Incidence rate, events per 1,000 person-years	HRs (95% CIs)		
				Model 1	Model 2	Model 3
BMI, kg/m^2^						
18.5	108	7288.2	14.82	0.67(0.553,0.811)	0.673(0.556,0.815)	0.651(0.538,0.788)
18.5–23.9	4162	183267.8	22.71	1(ref)	1(ref)	1(ref)
24–27.9	3884	120180.8	32.32	1.394(1.334,1.457)	1.387(1.327,1.449)	1.396(1.336,1.459)
≥ 28	1493	35766.8	41.74	1.773(1.671,1.882)	1.758(1.656,1.866)	1.769(1.666,1.878)
WC(cm)						
< 85(females) < 90(males)	5982	247788.5	24.14	1(ref)	1(ref)	1(ref)
≥ 85(females) ≥ 90(males)	3665	98715.0	37.13	1.515(1.453,1.58)	1.514(1.452,1.579)	1.513(1.45,1.578)
WtHR						
< 0.5	3415	151946.5	22.48	1(ref)	1(ref)	1(ref)
≥ 0.5	6232	194557.1	32.03	1.405(1.347,1.466)	1.408(1.35,1.469)	1.412(1.353,1.473)

Model 1: adjusted for age and gender; Model 2: adjusted for variables in Model 1 plus marital status, smoking, drinking, physical activity and RHR; Model 3: competing-risks survival regression adjusted covariates were the same as Model 2

*Abbreviations*: *OR* odd ratio, *CI* confidential interval, *BMI* body mass index, *WC* waist circumference, *WtHR* waist-to-height ratio, *HDC* hypertension–diabetes comorbidity

### Restricted cubic spline curves for three indicators and HDC risk

**Fig. 1:**
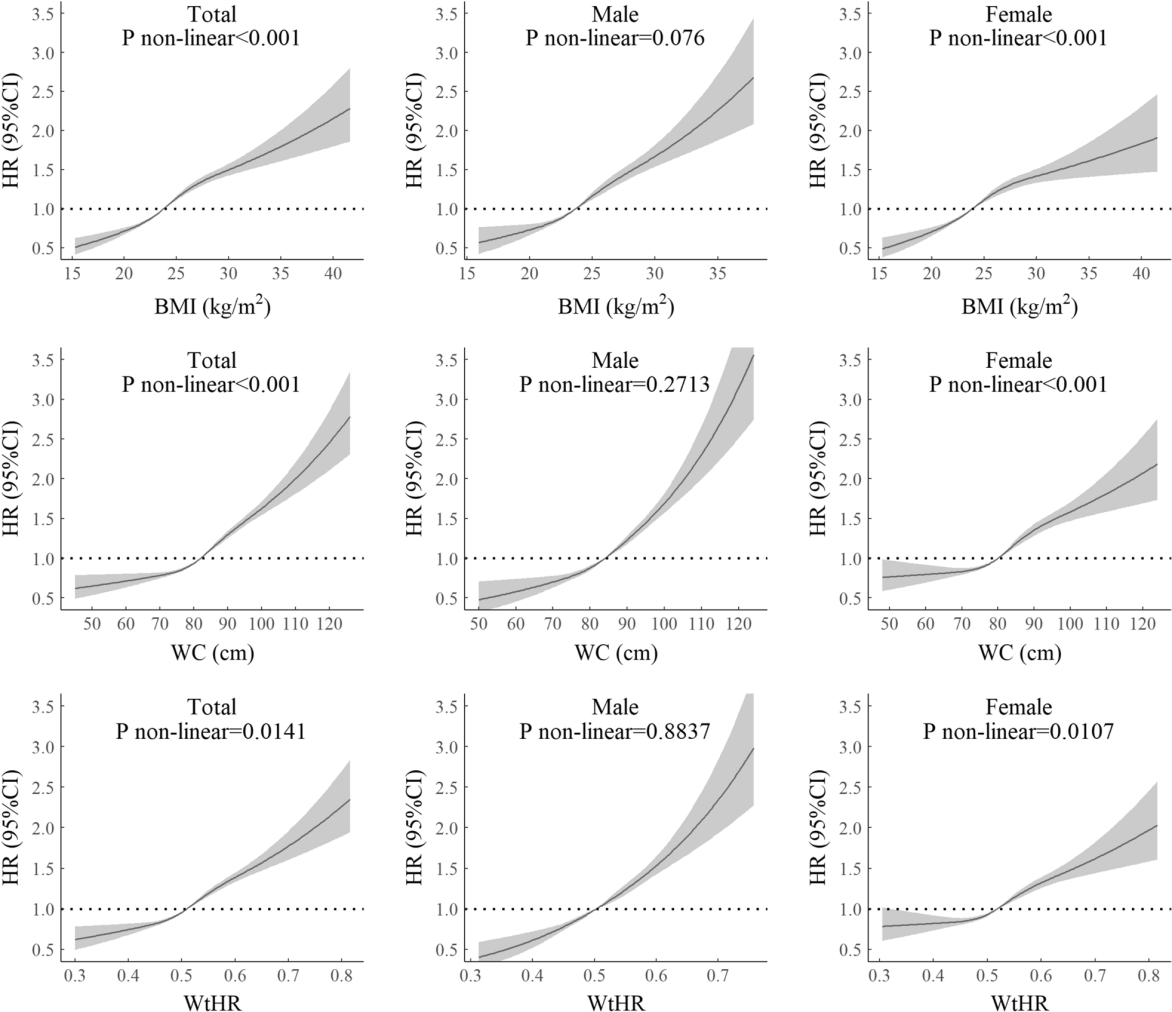
Relationship of BMI, WC and WtHR with the risk of HDC for all participants and subgroups of males and females. HRs are adjusted for age, sex (not for sex subgroup analysis), marital status, smoking, drinking, physical activity and RHR. Abbreviations: *OR* odd ratio; *CI* confidential interval; *BMI* body mass index; *WC* waist circumference; *WtHR* waist-to-height ratio; *HDC* hypertension–diabetes comorbidity

Figure 1 shows the spline curves for the association of HDC risk with the three tested indicators (BMI, WC and WtHR as a continuous variable). All of these associations exhibited a reverse J-shaped curve. The association between the three indicators and HDC morbidity indicated that the risks were increased when the three indicator tested values were increasing among all of the participants and the female subgroup. Additionally, BMI had a nonlinear association with HDC morbidity among all of the participants and female, based on the adjusted Cox model (P non-linear < 0.001), but the male subgroup had a similar curve with no significant nonlinear association (P non-linear = 0.076). WC had a nonlinear association with HDC morbidity among all participants and female (P non-linear < 0.001), but the male subgroup had a similar curve with no significant nonlinear association (P non-linear = 0.2713). Furthermore, WtHR had a nonlinear association with HDC morbidity among all of the participants (P non-linear = 0.0141) and females (P non-linear = 0.0107), but the male subgroup had a similar curve with no significant nonlinear association (P non-linear = 0.8837).

### Baseline BMI, WC and WtHR with respect to the risk of HDC

**Fig. 2:**
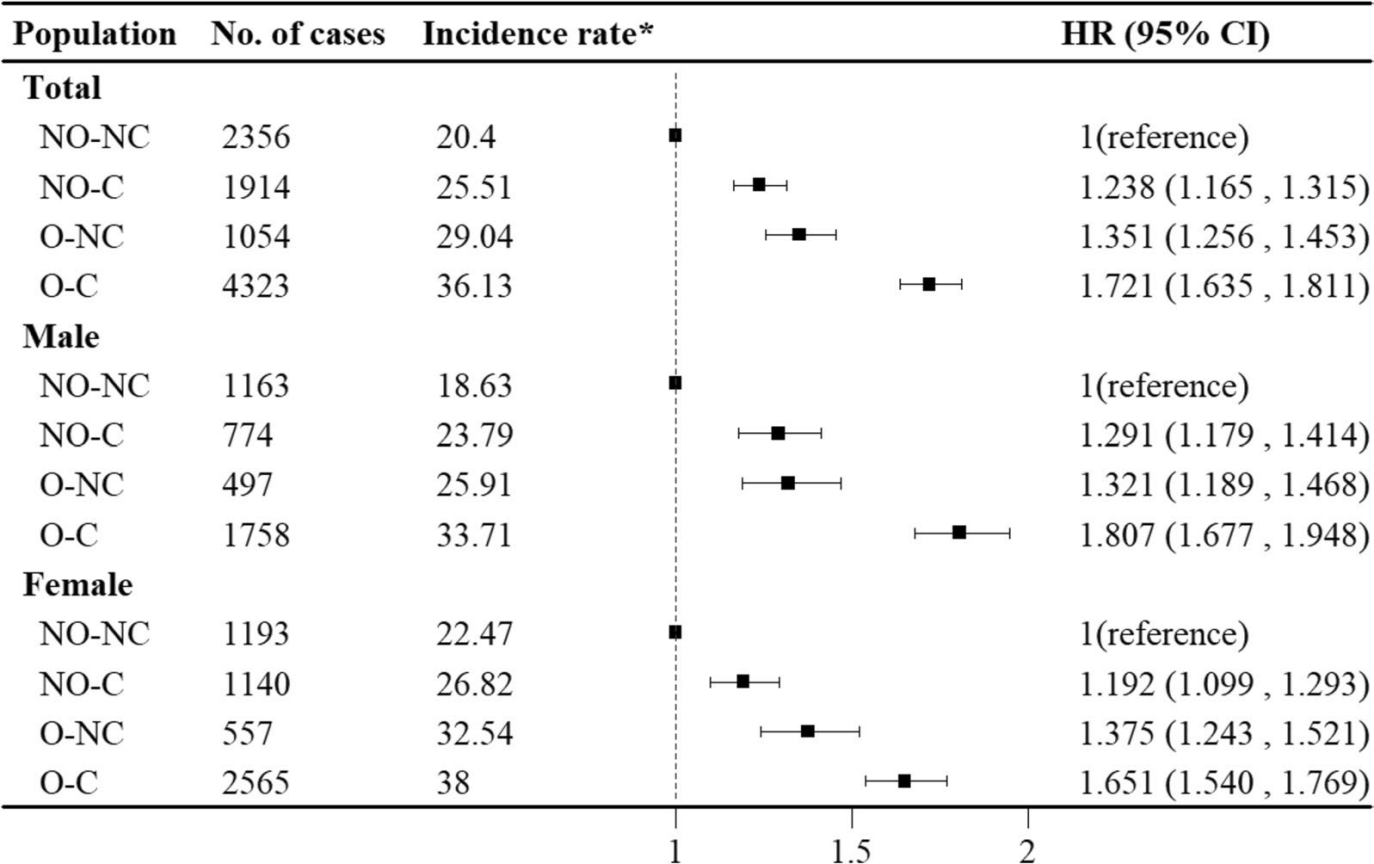
Combined effect of BMI, WC and WtHR with the risk of HDC for all participants and subgroups of males and females. HRs are adjusted for age, sex (not for sex subgroup analysis), marital status, smoking, drinking, physical activity and RHR. Abbreviations: NO nonoverweight (BMI < 24kg/m^2^); O overweight (BMI ≥ 24kg/m^2^); NC non-central obesity (WC < 90 in male/WC < 85 in female/WtHR < 0.5); C central obesity (WC ≥ 90 in male/WC ≥ 85 in female/WtHR ≥ 0.5); CI confdential interval; HR hazard ratio; * events per 1,000 person-years

The combined effects of baseline BMI, WC and WtHR on the risk of HDC are shown in Fig. 2. In total participants, the results showed that the additive interaction did exist (RERI: 0.131; 95% CI: 0.015 to 0.248); AP: 0.076; 95% CI: 0.008 to 0.144; S: 1.223; 95% CI: 1.002 to 1.493). Compared to the nonoverweight and noncentral obesity group, participants who were nonoverweight and who had central obesity (HR: 1.238; 95% CI:1.165, 1.315), participants who were overweight and who had noncentral obesity (HR: 1.351; 95% CI: 1.256, 1.453) or patients who were overweight and who had central obesity ((HR: 1.721; 95% CI: 1.635, 1.811) were at a significantly increased risk for HDC. In the subgroup analyses of sex, similar trends were also observed. In addition, we compared the subjects with similar high BMI and with or without high WC (Supplemental Fig. 2). Similar comparison was conducted between subjects with similar high WC with or without high BMI (Supplemental Fig. 3).

### Sensitivity analysis

We conducted three sensitivity analyses to assess the robustness of the results. In the sensitivity analysis 1, we excluded participants with hypertension or diabetes at baseline. In the sensitivity analysis 2, we excluded whose follow-up time was less than 2 year. In the sensitivity analysis 3, missing data were accounted for by multiple imputation. The results were observed to be robust in all the sensitivity analysis (Supplemental Tables 3-5). Supplemental Table 6 shows the baseline characteristics of the study population and deletion of missing data population.

## Discussion

In our study, the Cox proportional hazard regression was used to examine the relationship between obesity indicators and HDC in elderly individuals, and increased BMI, WC and WtHR were found to be associated with an increased risk of HDC. The same results were also observed in the sex subgroup analysis. The dose–response relationship that was analyzed by using restricted cubic splines demonstrated a nonlinear relationship between BMI, WC, WtHR and HDC among all of the participants and female subgroup, but the male subgroup had a similar curve with no significant nonlinear association. Furthermore, we found that WC and WtHR had similar predictive abilities in elderly individuals. When models were adjusted for multiple factors of age, sex, marital status, smoking, drinking, physical activity and RHR and were analyzed by using competing-risk survival regression, the results did not significantly change. Specifically, regardless of what covariates were adjusted in the models, the relationships of these anthropometric indicators with HDC always existed. Moreover, this study conducted an additive interaction analysis, which concluded a significant additive interaction between a general obesity indicator (BMI) and central obesity indicators (WC and WtHR), such that the incidence of HDC increased. To some extent, this study provides a better understanding of the association of anthropometric indicators of obesity with HDC, which may be more enlightening for HDC prevention.

Our study confirmed previous findings that both general and abdominal obesity conditions have strong and independent correlations with the development of HDC [13, 21–23]. Our study found that BMI, WC and WtHR were positively associated with HDC, and those positive associations were also observed in other studies. A cross-sectional study from the Centers for Disease Control and Prevention in Hubei Province, Central China (including 12,214 men and 13,142 women) reported that BMI and WtHR were strongly and significantly associated with the risk of HDC among Chinese adults [13]. Likewise, the Health Survey for England (HSE) analyzed nationally representative cross-sectional population surveys and found that generalized (BMI > 30kg/m^–2^) and abdominal obesity (WC > 102cm in men, > 88cm in women) were independently associated with the risk of hypertension, diabetes and HDC [21]. Furthermore, Heather P. Tarleton et al. analyzed cross-sectional data from the first round of the Los Angeles County Health and Nutrition Examination Survey and observed that BMI and WC were positively related to diabetes, hypertension and comorbidity in whites individuals, as well as the fact that African-Americans and BMI and WC were also positively related to diabetes and comorbidity in Hispanics [24]. In addition, we also found that being as lean as possible within the normal range of BMI, WC and WtHR may be a better suggestion in reducing the risks of HDC in elderly adults. Based on log-rank test, Kaplan–Meier curve was used to obtain the best cutoff value of continuous variable, so that the data on both sides of a point have the best difference. The appropriate cutoff points (Supplemental Table 7) for the prevention of HDC were 24 kg/m^2^ for BMI (which was consistent with the Chinese criteria of being overweight) and 0.5 for WtHR (which was consistent with previous studies in both men and women) [4, 20]. The optimal cutoff points for the prevention of HDC were 84 cm for WC in women and 83 cm for WC in men. Moreover, the values were slightly below the Chinese cutoffs for abdominal obesity and compared to previous studies [4, 19]. Differences in age and region may explain the discrepancy in the cutoff values.

Our study found that men were at a higher risk of developing HDC than women. Males and females exhibit differences in anatomical fat distribution, utilization of fat stores, levels of adipose tissue derived hormones and obesity comorbidities [25]. Previous studies have found that the distribution of body fat differs between men and women. Females are generally more likely to deposit fat both subcutaneously and on their lower extremities, whereas males are more likely to deposit fat in the abdominal region. The excessive accumulation of abdominal fat is associated with more health risks [26, 27]. The female pattern of fat distribution is conducive to improving cardiovascular risks at a similar BMI, and female fat distribution and expression regulation may be more genetically affected than males by environmental factors [28, 29]. In addition, many aspects of energy balance and glucose metabolism are differentially regulated in males and females, and males are more likely to develop IR than females [30, 31], which greatly increases the risk of HDC.

We also tested the joint effects of abdominal adiposity (as measured via WC and WtHR) and overall body adiposity (as measured via BMI). Some studies have suggested that joint indicators have a greater risk of health event outcomes [32, 33]. Other studies have suggested that substantial heterogeneity in the associations between obesity measures and diseases, gender, age and ethnic groups have different optimal indicators [34–36]. Our study found that the highest risk was observed in participants with overweight statuses combined with abdominal obesity in both the male and female subgroups. Although we observed that in the female subgroup, being overweight alone was associated with a higher risk of HDC than abdominal obesity alone, in the male subgroup, the risk of HDC was similar between being overweight alone and central obesity alone. Although a previous study suggested that there were significant correlations between BMI and WC, between BMI and WtHR and between WC and WtHR, BMI, WC and WtHR indices can be moderately and interchangeably used with caution [37]. Therefore, the concurrent determination of abdominal obesity and overall obesity may be beneficial for the accurate determination of future HDC risks.

There were several advantages of our study. First, to the best of our knowledge, our study was the first retrospective cohort study to explore the associations between anthropometric indicators of obesity and HDC among older people with a large-scale health check in central China, and the sample size and statistical power were adequate. It is of practical significance to improve upon the relevant research. Second, standardized measurements were used in this study, and an annual health examination dataset was used to avoid discrepancies between participants’ self-reports and the actual situation. Third, we also conducted sensitivity analysis to assess the robustness of the association between the three indicators and the incidence of HDC risk.

However, some limitations of this study should be noted. First, the subjects of this study were primarily elderly people in Xinzheng, Henan Province, Central China, which limits the universality of this study; therefore, we could not compare the relationship between obesity and HDC in other age groups or the predictive ability of these indicators for HDC. Second, we adjusted for major confounders in the analysis, but some potential factors may exist that we did not adjust for, including unmeasured factors such as genetic factors and diet. Third, due to the fact that our study sample limits the generality of our results, the dose–response association should be cautiously considered. In the future, more studies may be needed to analyze the dose–response relationship between obesity indicators and HDC to further verify this result and to improve upon the accuracy of our study.

## Conclusions

Overall, this study found that increased BMI, WC and WtHR were associated with an increased risk of HDC. The same results were found in the sex subgroup analysis. Moreover, there was an additive interaction between general body adiposity (as measured via BMI) and central obesity (as measured via WC and WtHR) for HDC. The concurrent determination of abdominal obesity and overall obesity may be beneficial for the accurate determination of future HDC risk. Therefore, the reasonable control of BMI, WC and WtHR may be an effective measure to prevent HDC among elderly individuals.

### Supplementary Information

**Additional file 1: Supplementary Figure 1.** Screening flowchart of participants. **Supplementary Table 1.** The follow-up and complete cases of cohort every year. **Supplementary Table 2.** The AICs of different knots in restricted cubic spline analysis. **Supplementary Figure 2.** Combined effect of BMI, WC and WtHR with the risk of HDC for all participants and subgroups of males and females. **Supplementary Figure 3.** Combined effect of BMI, WC and WtHR with the risk of HDC for all participants and subgroups of males and females. **Supplementary Table 3.** Sensitive analysis 1 of BMI, WC and WtHR with HDC risk. **Supplementary Table 4.** Sensitive analysis 2 of BMI, WC and WtHR with HDC risk. **Supplementary Table 5.** Sensitive analysis 3 of BMI, WC and WtHR with HDC risk after multiple imputation. **Supplementary Table 6.** Baseline characteristics of the study population and deletion of missing data population. **Supplementary Table 7.** The best cutoffs of BMI, WC and WtHR with HDC risk. 

## Data Availability

The datasets generated and/or analyzed during the current study are not publicly available due to confidentiality requirements of third parties, but are available from the corresponding author on request.

## Abbreviations

BMI: Body Mass Index
WC: Waist Circumference
WtHR: Waist-to-Height Ratio
HDC: Hypertension–diabetes comorbidity
HR: Hazard ratio
CI: Confidence Interval
RHR: Resting Heart Rate
SD: Standard Deviation
RERI: Relative Excess Risk due to Interaction
AP: Attributable Proportion due to interaction
S: Synergy index
