# Supplementary material for: Relationship between obesity indicators and hypertension–diabetes comorbidity in an elderly population: a retrospective cohort study
Source: BMC Geriatr. 2023 Nov 30;23:789. doi: 10.1186/s12877-023-04510-z (PMC10691080; doi:10.1186/s12877-023-04510-z)
Supplement: Supplementary file 1 — Additional file 1: Supplementary Figure 1. Screening flowchart of participants. Supplementary Table 1. The follow-up and complete cases of cohort every year. Supplementary Table 2. The AICs of different knots in restricted cubic spline analysis. Supplementary Figure 2. Combined effect of BMI, WC and WtHR with the risk of HDC for all participants and subgroups of males and females. Supplementary Figure 3. Combined effect of BMI, WC and WtHR with the risk of HDC for all participants and subgroups of males and females. Supplementary Table 3. Sensitive analysis 1 of BMI, WC and WtHR with HDC risk. Supplementary Table 4. Sensitive analysis 2 of BMI, WC and WtHR with HDC risk. Supplementary Table 5. Sensitive analysis 3 of BMI, WC and WtHR with HDC risk after multiple imputation. Supplementary Table 6. Baseline characteristics of the study population and deletion of missing data population. Supplementary Table 7. The best cutoffs of BMI, WC and WtHR with HDC risk. [file 12877_2023_4510_MOESM1_ESM.docx]

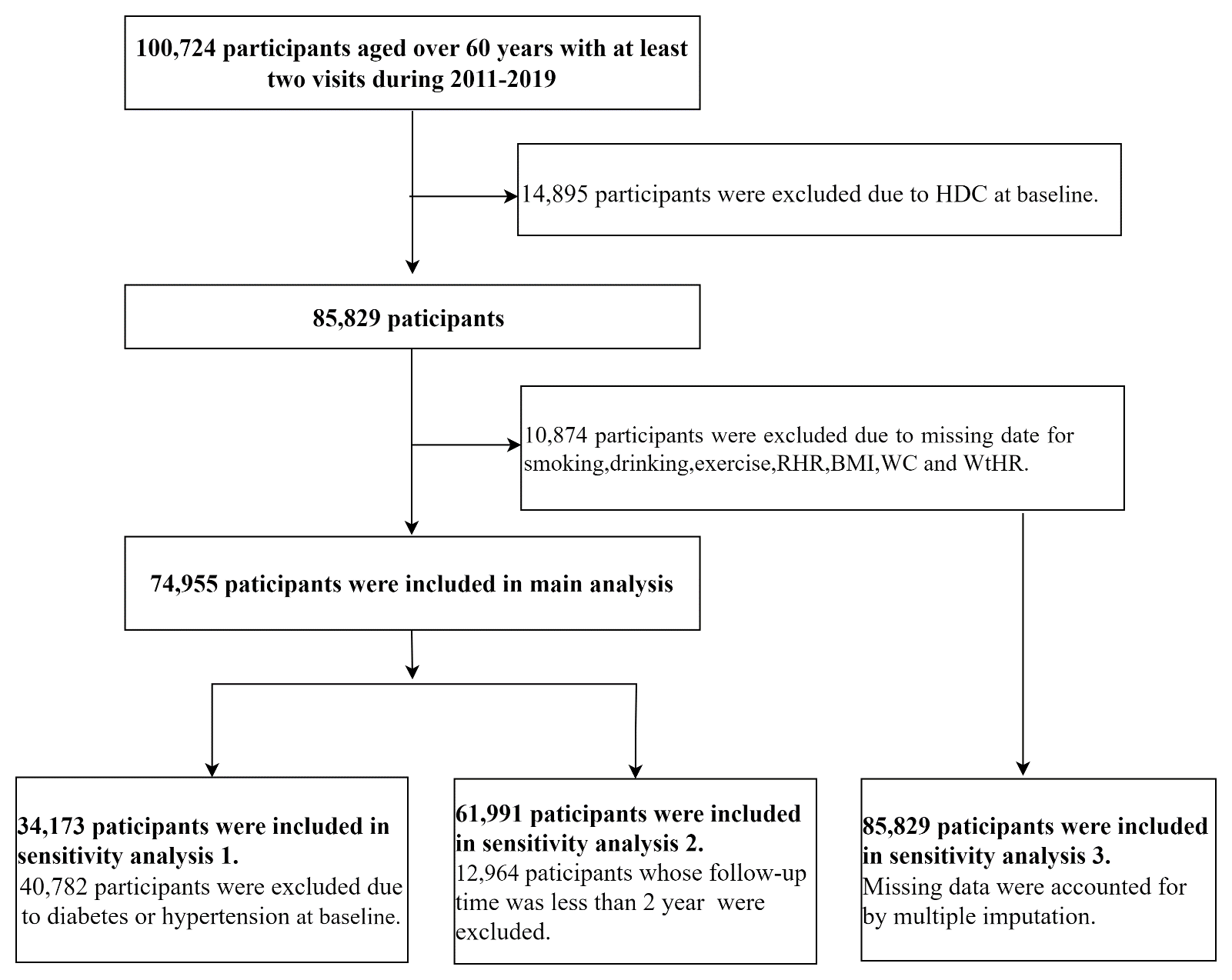


Supplementary Figure 1. Screening flowchart of participants

Supplementary Table 1. The follow-up and complete cases of cohort every year

| Time | Population of cohort | | HDC of cohort | | Death of cohort | | Death of cohort before HDC | |
| --- | --- | --- | --- | --- | --- | --- | --- | --- |
|  | number | Percent (%) | number | Percent (%) | number | Percent (%) | number | Percent (%) |
| 2011 | 22890 | 30.54 |  |  |  |  |  |  |
| 2012 | 10655 | 14.22 | 305 | 3.16 | 74 | 0.84 | 74 | 0.92 |
| 2013 | 16941 | 22.6 | 1045 | 10.83 | 634 | 7.16 | 612 | 7.62 |
| 2014 | 2588 | 3.45 | 807 | 8.37 | 1089 | 12.29 | 1035 | 12.88 |
| 2015 | 8908 | 11.88 | 1209 | 12.53 | 1226 | 13.84 | 1147 | 14.28 |
| 2016 | 1927 | 2.57 | 1083 | 11.23 | 1478 | 16.68 | 1349 | 16.79 |
| 2017 | 7612 | 10.16 | 1748 | 18.12 | 1493 | 16.85 | 1328 | 16.53 |
| 2018 | 3434 | 4.58 | 1209 | 12.53 | 1655 | 18.68 | 1455 | 18.11 |
| 2019 |  |  | 2241 | 23.23 | 1210 | 13.66 | 1033 | 12.86 |
| Total | 74955 | 100 | 9647 | 100 | 8859 | 100 | 8033 | 100 |

Supplementary Table 2. The AICs of different knots in restricted cubic spline analysis

|  |  | AIC | | |
| --- | --- | --- | --- | --- |
| Variables | Knots | Total | Men | Women |
| BMI | 3 | 204607.8 | 82652.65 | 108738.7 |
|  | **4** | **204598.8** | **82650.76** | **108735.8** |
|  | 5 | 204599.8 | 82652.58 | 108737 |
|  | 6 | 204600.4 | 82653.91 | 108738.9 |
|  | 7 | 204601.1 | 82653.95 | 108741.1 |
| WC | 3 | 204619 | 82611.12 | 108784.4 |
|  | **4** | **204608.3** | **82611.93** | **108772.5** |
|  | 5 | 204609.8 | 82614.05 | 108772.2 |
|  | 6 | 204609.6 | 82612.8 | 108765.8 |
|  | 7 | 204609.2 | 82614.57 | 108768.6 |
| WtHR | 3 | 204746.7 | 82672.29 | 108839.9 |
|  | **4** | **204740.2** | **82674.25** | **108833.7** |
|  | 5 | 204741.8 | 82675.65 | 108835.1 |
|  | 6 | 204742.9 | 82675 | 108834.4 |
|  | 7 | 204742.9 | 82678.04 | 108836 |


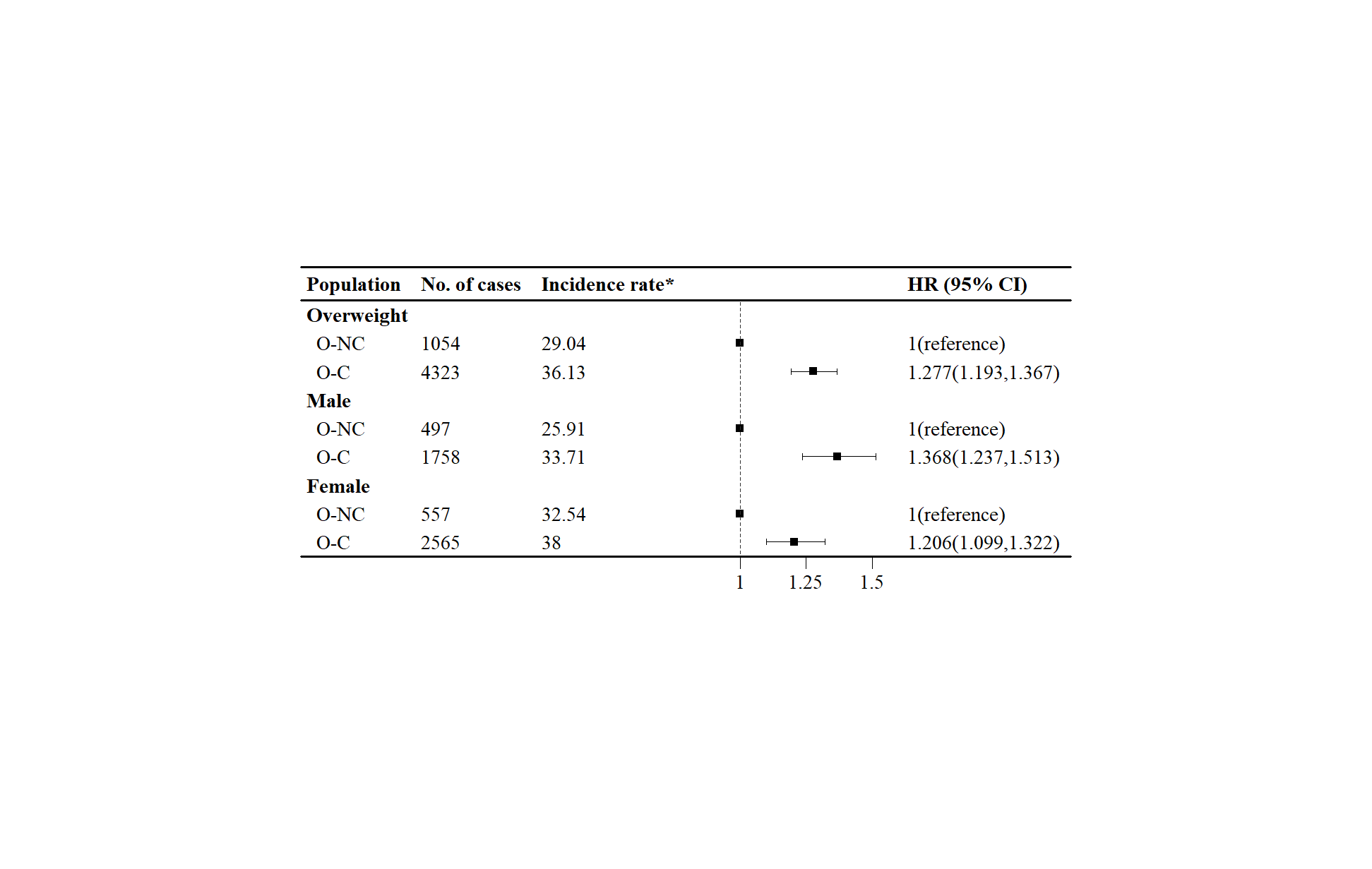


Supplementary Figure 2. Combined effect of BMI, WC and WtHR with the risk of HDC for all participants and subgroups of males and females.


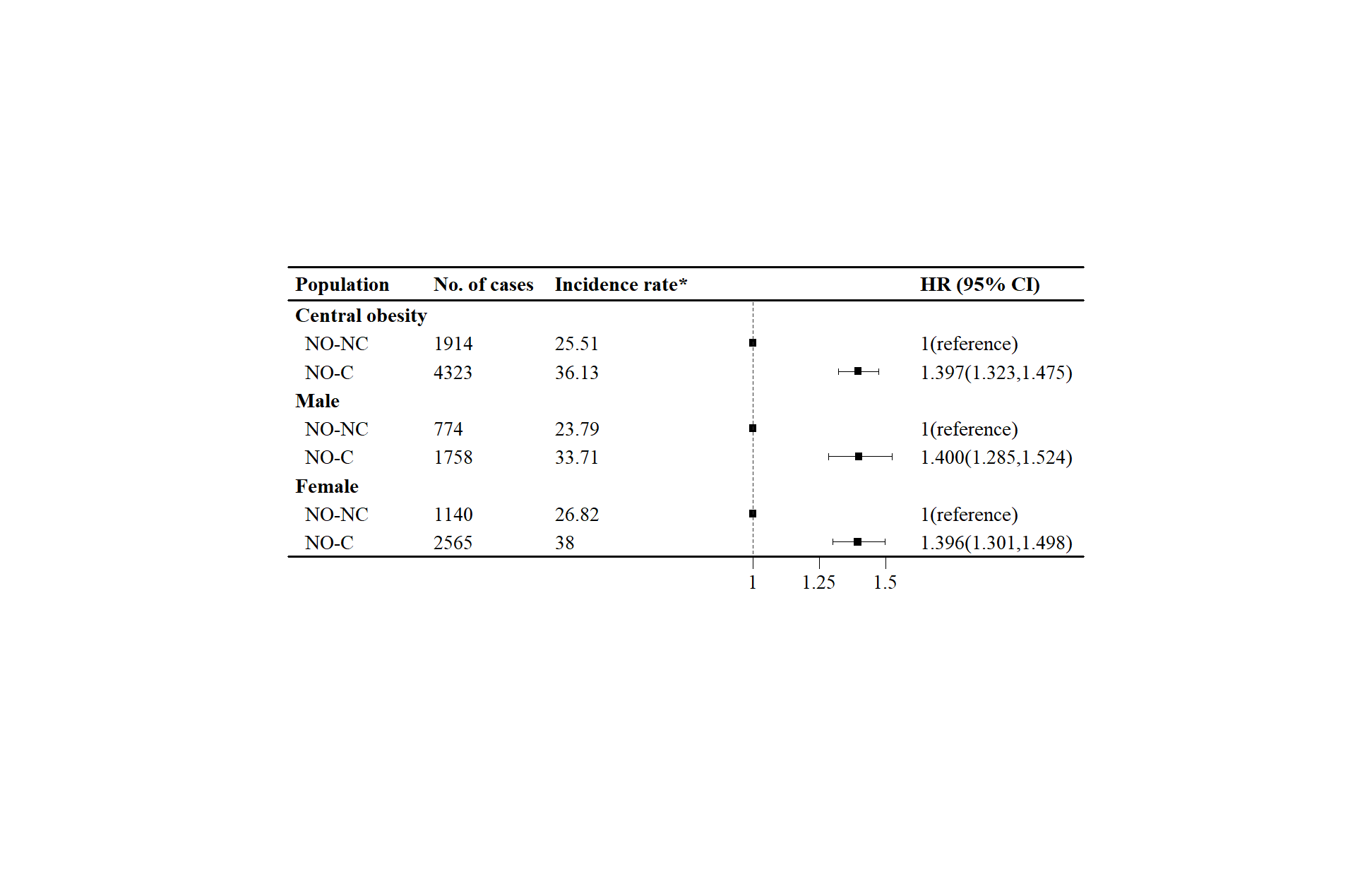


Supplementary Figure 3. Combined effect of BMI, WC and WtHR with the risk of HDC for all participants and subgroups of males and females.

Supplementary Table 3. Sensitive analysis 1 of BMI, WC and WtHR with HDC risk

| Variables |  | No. of cases | No. of person-years | Incidence rate, events per 1,000 person-years | HRs (95% CIs) | | |
| --- | --- | --- | --- | --- | --- | --- | --- |
|  |  |  |  |  | Model 1 | Model 2 | Model 3 |
| BMI, kg/m^2^ | <18.5 | 25 | 3963 | 6.31 | 0.659(0.442,0.98) | 0.66(0.443,0.982) | 0.63(0.424,0.937) |
|  | 18.5-23.9 | 941 | 95220 | 9.88 | 1(ref) | 1(ref) | 1(ref) |
|  | 24-27.9 | 722 | 49403 | 14.61 | 1.474(1.337,1.625) | 1.469(1.332,1.62) | 1.481(1.343,1.632) |
|  | ≥28 | 197 | 10268 | 19.19 | 1.957(1.676,2.284) | 1.946(1.666,2.272) | 1.954(1.672,2.284) |
| WC (cm) | <85(females) <90(males) | 1303 | 122730 | 10.62 | 1(ref) | 1(ref) | 1(ref)) |
|  | ≥85(females)  ≥90(males) | 582 | 36123 | 16.11 | 1.543(1.397,1.704) | 1.54(1.394,1.702) | 1.54(1.393,1.703) |
| WtHR | <0.5 | 809 | 79523 | 10.17 | 1(ref) | 1(ref) | 1(ref) |
|  | ≥0.5 | 1076 | 79330 | 13.56 | 1.34(1.222,1.469) | 1.341(1.222,1.471) | 1.343(1.224,1.473) |

Supplementary Table 4. Sensitive analysis 2 of BMI, WC and WtHR with HDC risk

| Variables |  | No. of cases | No. of person-years | Incidence rate, events per 1,000 person-years | HRs (95% CIs) | | |
| --- | --- | --- | --- | --- | --- | --- | --- |
|  |  |  |  |  | Model 1 | Model 2 | Model 3 |
| BMI, kg/m^2^ | <18.5 | 75 | 6940 | 10.81 | 0.677(0.538,0.851) | 0.68(0.54,0.855) | 0.654(0.521,0.822) |
|  | 18.5-23.9 | 2919 | 175005 | 16.68 | 1(ref) | 1(ref) | 1(ref) |
|  | 24-27.9 | 2698 | 113599 | 23.75 | 1.391(1.32,1.467) | 1.384(1.313,1.459) | 1.396(1.325,1.472) |
|  | ≥28 | 1025 | 33407 | 30.68 | 1.788(1.665,1.921) | 1.774(1.651,1.906) | 1.788(1.663,1.923) |
| WC (cm) | <85(females) <90(males) | 4273 | 236869 | 18.04 | 1(ref) | 1(ref) | 1(ref) |
|  | ≥85(females)  ≥90(males) | 2444 | 92081 | 26.54 | 1.487(1.414,1.563) | 1.485(1.411,1.562) | 1.487(1.413,1.565) |
| WtHR | <0.5 | 2441 | 145850 | 16.74 | 1(ref) | 1(ref) | 1(ref) |
|  | ≥0.5 | 4276 | 183100 | 23.35 | 1.404(1.335,1.476) | 1.405(1.336,1.477) | 1.413(1.344,1.486) |

Supplementary Table 5. Sensitive analysis 3 of BMI, WC and WtHR with HDC risk after multiple imputation

| Variables |  | No. of cases | No. of person-years | Incidence rate, events per 1,000 person-years | HRs (95% CIs) | | |
| --- | --- | --- | --- | --- | --- | --- | --- |
|  |  |  |  |  | Model 1 | Model 2 | Model 3 |
| BMI, kg/m^2^ | <18.5 | 139 | 9131 | 15.22 | 0.683(0.577,0.809) | 0.686(0.579,0.812) | 0.661(0.559,0.782) |
|  | 18.5-23.9 | 4891 | 214545 | 22.8 | 1(ref) | 1(ref) | 1(ref) |
|  | 24-27.9 | 4520 | 141335 | 31.98 | 1.375(1.321,1.432) | 1.368(1.314,1.425) | 1.379(1.324,1.436) |
|  | ≥28 | 1777 | 42353 | 41.96 | 1.773(1.679,1.873) | 1.759(1.665,1.858) | 1.77(1.676,1.871) |
| WC (cm) | <85(females) <90(males) | 7175 | 293765 | 24.42 | 1(ref) | 1(ref) | 1(ref) |
|  | ≥85(females)  ≥90(males) | 4152 | 113599 | 36.55 | 1.472(1.416,1.53) | 1.469(1.413,1.527) | 1.467(1.41,1.525) |
| WtHR | <0.5 | 4012 | 174893 | 22.94 | 1(ref) | 1(ref) | 1(ref) |
|  | ≥0.5 | 7315 | 232471 | 31.47 | 1.349(1.297,1.402) | 1.35(1.298,1.403) | 1.354(1.302,1.408) |

Supplementary Table 6. Baseline characteristics of the study population and deletion of missing data population

|  |  | Missing data | |  |
| --- | --- | --- | --- | --- |
| Characteristics | Total  (n = 85,829) | Yes  (n = 10,874) | No  (n = 74,955) | P value |
| Age (years) | 66.98 (7.46) | 69.07 (7.61) | 66.68 (7.39) | <0.001 |
| Gender (%) |  |  |  | 0.956 |
| Men | 41069 (47.85) | 5200 (47.82) | 35869 (47.85) |  |
| Women | 44760 (52.15) | 5674 (52.18) | 39086 (52.15) |  |
| Marital status (%) |  |  |  | <0.001 |
| Living with partner | 68525 (79.84) | 8162 (75.06) | 60363 (80.53) |  |
| Living without partner | 17304 (20.16) | 2712 (24.94) | 14592 (19.47) |  |
| Smoking (%) |  |  |  | <0.001 |
| Never | 73811 (86.42) | 9877 (94.49) | 63934 (85.30) |  |
| Current or previous | 11597 (13.58) | 576 (5.51) | 11021 (14.70) |  |
| Drinking (%) |  |  |  | <0.001 |
| Never | 79679 (93.41) | 10118 (97.80) | 69561 (92.80) |  |
| Occasionally | 3267 (3.83) | 154 (1.49) | 3113 (4.15) |  |
| frequently | 698 (0.82) | 15 (0.14) | 683 (0.91) |  |
| Daily | 1657 (1.94) | 59 (0.57) | 1598 (2.13) |  |
| Physicial activity (%) |  |  |  | <0.001 |
| Never | 65967 (77.09) | 9346 (88.00) | 56621 (75.54) |  |
| Occasionally | 3914 (4.57) | 309 (2.91) | 3605 (4.81) |  |
| More than once a week | 3392 (3.96) | 342 (3.22) | 3050 (4.07) |  |
| Daily | 12302 (14.38) | 623 (5.87) | 11679 (15.58) |  |
| BMI(kg/m^2^) |  |  |  | <0.001 |
| normal | 43997 (51.44) | 5453 (51.54) | 38544 (51.42) |  |
| underweight | 1867 (2.18) | 338 (3.19) | 1529 (2.04) |  |
| overweight | 30154 (35.25) | 3626 (34.27) | 26528 (35.39) |  |
| obesity | 9518 (11.13) | 1164 (11.00) | 8354 (11.15) |  |
| WC(cm) |  |  |  | <0.001 |
| normal waist | 53839 (68.83) | 2356 (72.25) | 51483 (68.69) |  |
| central obesity | 24377 (31.17) | 905 (27.75) | 23472 (31.31) |  |
| WtHR |  |  |  | <0.001 |
| normal | 32348 (41.36) | 1452 (44.64) | 30896 (41.22) |  |
| central obesity | 45860 (58.64) | 1801 (55.36) | 44059 (58.78) |  |
| RHR(beats/min) | 73.54 (8.26) | 72.80 (9.74) | 73.59 (8.14) | <0.001 |

Supplementary Table 7. The best cutoffs of BMI, WC and WtHR with HDC risk

| variables | cutoff | | |
| --- | --- | --- | --- |
|  | Total | Men | Women |
| BMI | 24.20307 | 24.09297 | 24.54888 |
| WC | 83.2 | 83 | 84 |
| WtHR | 0.545455 | 0.519737 | 0.545455 |
